# Policy and Perception: Comparing Attitudes Toward LGBTQ and Gender Equality Between Long-Term Care Staff in Taiwan and Indonesia

**DOI:** 10.1097/jnr.0000000000000759

**Published:** 2026-08-13

**Authors:** Fika Lilik INDRAWATI, Chia-Jung HSIEH

**Affiliations:** 1 Professional Midwife Education Program, Faculty of Health Science, Universitas Respati Yogyakarta, Yogyakarta, Indonesia; 2 School of Nursing, College of Nursing, National Taipei University of Nursing and Health Sciences, Taipei, Taiwan

**Keywords:** LGBTQ policies, gender equality, long-term care, health care staff attitudes

## Abstract

**Background:**

Integrating lesbian, gay, bisexual, transgender, and queer (LGBTQ) policies within health care is critical to ensuring equitable treatment and improved health outcomes for LGBTQ individuals. However, significant disparities remain in the implementation of these policies, with substantial challenges persisting in culturally and legally diverse settings such as Indonesia and Taiwan. This study explores these issues from the perspectives of long-term care staff in these two countries, focusing on their attitudes towards LGBTQ individuals and the effectiveness of existing policies.

**Purpose:**

The primary objective of this study was to compare the implementation of LGBTQ policies with attitudes towards gender equality among long-term care staff in Indonesia and Taiwan. The secondary objective was to develop effective strategies for creating inclusive and equitable health care environments in diverse cultural contexts.

**Methods:**

A mixed-methods approach using both qualitative and quantitative research methods was employed, and a convenience sampling method was used to select long-term care facilities in both countries. The targeted facilities included small-to-medium-sized centres located in urban and semi-urban regions. One hundred and ninety-seven long-term care staff (Taiwan = 135 and Indonesia = 62) completed the questionnaire. Data were collected using a structured survey designed to measure attitudes toward LGBTQ policies and gender equality using Likert-scale items. Descriptive and inferential statistics, including percentages, means, and independent *t* test, were used to analyse the quantitative data. For the qualitative component, the policy formation process was analysed based on the policy concept of Longest (2006) to provide further context to the findings.

**Results:**

The analysis revealed significant differences in attitudes toward homosexuality (*t* = 19.81, *p* = .001) and transgender individuals (*t* = 7.12, *p* = .001) between the Taiwanese and Indonesian participants, with the Taiwanese participants demonstrating higher acceptance of homosexuality (*M* = 18.26, *SD* = 4.90) and transgender individuals (*M* = 21.90, *SD* = 4.07) than their Indonesian peers (*M* = 9.03, *SD* = 1.55 and *M* = 18.42, *SD* = 2.67, respectively). The results also indicate the supportive legal environment in Taiwan facilitates more progressive policies, while the conservative cultural norms and lack of legal protections in Indonesia create substantial barriers to LGBTQ inclusivity in long-term care settings.

**Conclusions:**

The findings highlight the critical role of supportive political climates and comprehensive policy frameworks in advancing LGBTQ rights within health care settings. While Taiwan has made significant progress, ongoing efforts are needed to bridge the gap between policy and practice. In Indonesia, substantial changes in the political and cultural landscape are critical to advancing LGBTQ inclusivity in health care. The findings of this study underscore the need for culturally sensitive strategies that address both legal and societal challenges to create a truly inclusive environment for LGBTQ individuals across diverse health care settings.

## Introduction

The global health care landscape is undergoing a significant transformation, with increasing emphasis given to inclusivity for lesbian, gay, bisexual, transgender, and queer (LGBTQ) individuals. Recent research highlights that training programs tailored to specific care needs improve patient satisfaction and overall health outcomes (Park & Hwang, 2024).

Countries and regions with protective policies (e.g., nondiscrimination laws) have widely shown significant improvements in health care access and quality for LGBTQ individuals. State-level protective policies, for example, have been associated with better health outcomes in transgender populations (King et al., 2024). Conversely, countries and regions in which LGBTQ identities are criminalized or stigmatized generally severely limit health care access for this population, leading to poorer health outcomes (Tan, 2024). The interplay between cultural acceptance and legal protection significantly shapes the health care experiences of LGBTQ individuals, with supportive environments facilitating better health service utilization (Green et al., 2022; Turpin et al., 2022).

Enacting inclusive LGBTQ policies in long-term care facilities is known to have a profound impact on the health experiences and care outcomes of LGBTQ individuals, with findings showing these policies enhance the quality of care for LGBTQ residents and improve mental health outcomes. Specifically, these policies help reduce perceived isolation and discrimination, which are both critical factors influencing overall well-being (Hurd & Li, 2024; Singh et al., 2024). Also, implementing LGBTQ-affirming care practices mitigates the adverse effects of minority stress, leading to better health outcomes (Eschliman et al., 2023; Singh et al., 2024).

Despite these advancements, staff attitudes towards LGBTQ individuals remain a crucial determinant of care quality in health care settings. Negative attitudes and biases may lead to/exacerbate discriminatory practices that significantly affect patient experiences and health outcomes. Conversely, when health care staff are trained in LGBTQ inclusivity and demonstrate positive attitudes, patients are more likely to engage with health care services and achieve better health outcomes (Keuroghlian et al., 2021).

Disparities in LGBTQ health care policies are particularly pronounced in Asia, where legal and social challenges hinder access to health care. Countries like Malaysia and Indonesia maintain laws that criminalize same-sex relationships, deterring LGBTQ individuals from seeking care (Tan, 2024). In contrast, Taiwan has made strides in LGBTQ rights, fostering a more inclusive health care environment (Feroe et al., 2022).

Taiwan is the only country in Asia that legally recognizes and promotes LGBTQ rights (Chen et al., 2019). By contrast, Indonesia, a country in which 86% of the population identifies as Muslim, largely follows religious doctrines that reject homosexuality and LGBTQ identities (Djaja & Nisa, 2023; Hidayat & Darmadi, 2019). Recent legislative changes in Taiwan and Indonesia have significantly impacted public and professional attitudes towards LGBTQ rights. Taiwan’s legalization of same-sex marriage in 2019 increased visibility and acceptance, encouraging inclusive health care practices (Feroe et al., 2022). Conversely, the lack of legal protections and rising anti-LGBTQ sentiment in Indonesia perpetuate discrimination, which adversely affects LGBTQ health outcomes (Tan, 2024). These contrasting frameworks illustrate how legal contexts shape societal attitudes and LGBTQ well-being.

The aim of this study was to compare LGBTQ policies and staff attitudes toward gender equality in Indonesia and Taiwan and then, based on the results, suggest strategies for promoting and realizing more inclusive health care environments. This cross-cultural research was intended to highlight how cultural and legal contexts shape the implementation and effectiveness of LGBTQ-inclusive policies, thus contributing to the broader discourse on health care inclusivity and informing global policy development.

## Methods

### Design, Setting, Sampling

Both qualitative and quantitative approaches were employed in this mixed-methods study to investigate LGBTQ policies and attitudes toward gender equality among long-term care staff in Indonesia and Taiwan. Study data were collected between November and December 2023. A convenience sampling method was used to select the long-term care facilities targeted, which included small-to-medium-sized facilities in urban and semiurban areas of Taiwan and Indonesia. In Indonesia, most of the targeted facilities were in the provinces of Central and East Java where staff are predominantly Muslim. The final sample included 197 staff (135 from Taiwan and 62 from Indonesia), all of whom completed the study survey.

### Instrument

The quantitative component involved structured questionnaires using two primary tools: the Homosexual Attitudes Scale (HAS) and Transgender Awareness Scale. The HAS, developed by Kite and Deaux (1986), is designed to assess attitudes toward homosexuality using six items scored on a Likert Scale. These items gauge views on homosexuality, with responses ranging from *strongly agree* (1) to *strongly disagree* (4). The total possible scale score ranges from 6 to 24, with higher scores indicating more positive attitudes (Executive Yuan Gender Equality Council, 2020). The HAS has previously demonstrated high internal consistency (alpha coefficient = .92) and moderate test-retest reliability (.71). The Transgender Awareness Scale is designed to assess opinions on transgender issues using eight items, with a total possible scale score ranging from 8 to 32. Both of the questionnaires were translated into Bahasa Indonesia using forward-backward translation, then reviewed by experts for content validity. To ensure clarity and cultural appropriateness, the translated version was pilot-tested on five long-term care staff in Indonesia who were not part of the main study sample. During this pilot study, the five staff were asked to complete the survey and provide feedback on the wording, comprehension, and relevance of each item. Minor revisions were made based on their feedback to improve linguistic clarity and ensure all questions were easily understood and contextually appropriate. The survey was administered by trained research assistants familiar with local contexts.

### Data Collection

The study survey was administered online using Google Forms. The research assistants distributed a link to the survey to staff in the selected long-term care facilities through institutional WhatsApp groups. An introductory message explained the study objectives, and electronic informed consent was obtained before participants were allowed to access the questionnaire. Participation was voluntary and anonymous. All of the participants completed the online questionnaire on either a personal or institutional device. To support the participants during data collection in case of technical issues or clarification needs, contact information for the local research coordinators was provided. The online format enabled broader geographic reach and ensured confidentiality in both countries.

### Data Analysis

Data analysis was performed using IBM SPSS Statistics Version 22 (IBM Corp., Armonk, NY, USA). An independent *t* test compared mean scores between groups. For qualitative analysis, the policy formation process of Longest (2006) was used to provide context to the quantitative findings.

### Ethical Considerations

The study was conducted in accordance with the ethical guidelines and principles of the Declaration of Helsinki. All subjects gave informed written consent for the experimental procedures, which were approved by the institutional review board of Fu Jen Catholic University (FJU-IRN NO: C112110).

## Results

### Participant Demographic Characteristics

The demographic profile of the participants (*N* = 197) showed significant differences between the Taiwanese (*n* = 135) and Indonesian (*n* = 62) subgroups. A notable gender disparity was observed, with females accounting for 93.5% and 83.0%, respectively, of the Indonesian and Taiwanese subgroups. The Taiwanese participants were older (*M* = 46.3, *SD* = 13.2) than their Indonesian counterparts (*M* = 36.5, *SD* = 8.8). Education levels also differed significantly, with 82.3% of the Indonesian subgroup reporting high school as their highest level of formal education, compared with 26.7% in the Taiwanese subgroup (Table 1).

**Table 1:** Participant Demographic Characteristics

Variable	All (*N*=197)	Taiwan (*n*=135)	Indonesia (*n*=62)
	*n* (%)	*n* (%)	*n* (%)
Gender			
Male	27 (13.7)	23 (17.0)	4 (6.5)
Female	170 (86.3)	112 (83.0)	58 (93.5)
Age (year; *M* and *SD*)	43.21 (12.8)	46.3 (13.2)	36.5 (8.8)
Work experience (year; *M* and *SD*)	12.26 (11.2)	14.9 (12.5)	6.6 (3.3)
Educational level			
Junior high school	18 (9.1)	10 (7.4)	8 (12.9)
High school/vocational	87 (44.2)	36 (26.7)	51 (82.3)
College or University	80 (40.6)	77 (57.0)	3 (4.8)
Graduate school	12 (6.1)	12 (8.9)	0 (0.0)
Previous experience interacting with LGBTQ individuals			
No experience	62 (31.5)	44 (32.6)	18 (29.0)
Experienced	109 (55.3)	75 (55.6)	34 (54.8)
Uncertainty	26 (13.2)	16 (11.9)	10 (16.1)

The univariate and comparison analyses of attitudes toward homosexuality and transgender individuals between the Taiwanese and Indonesian participants revealed significant differences. The Taiwanese subgroup demonstrated significantly more positive attitudes towards homosexuality (*M* = 18.26, *SD* = 4.90) and transgender individuals (*M* = 21.90, *SD* = 4.07) than their Indonesian counterparts (*M* = 9.03, *SD* = 1.55 and *M* = 18.42, *SD* = 2.67, respectively; all *p values* = .001; Table 2).

**Table 2:** Univariate Comparison of Attitudes Toward Homosexual and Transgender Individuals Between Countries

Variable	All	Taiwan	Indonesia	*t*	*p*
	Mean (*SD*)	Mean (*SD*)	Mean (*SD*)		
Homosexual attitude	15.36 (5.9)	18.26 (4.90)	9.03 (1.55)	19.81	.001
Transgender attitude	20.80 (4.0)	21.90 (4.07)	18.42 (2.67)	7.12	.001

The analysis of the policy environments in both countries revealed differences with regard to approaches to LGBTQ inclusivity in long-term care settings. The supportive political climate in Taiwan has facilitated the development and implementation of policies designed to protect LGBTQ individuals, while the more resistant climate in Indonesia necessitates reliance on strategic alliances and international advocacy to promote inclusivity (Table 3). These findings highlight the importance of tailoring policy strategies to the specific political and cultural contexts of each country.

**Table 3:** LGBTQ Policies Between Taiwan and Indonesia

Dimension	Similarities	
	Taiwan	Indonesia
Problem identification	Both countries face significant discrimination against LGBTQ individuals in long-term care settings	
Solution exploration	Both have initiated training programs for caregivers to reduce discrimination	

Dimension	Differences	
	Taiwan	Indonesia
Policy environment	Taiwan has more supportive policies and a favorable political climate	Indonesia lacks protective policies and faces strong opposition
Political support	Taiwan benefits from strong political and public support	Indonesia contends with significant conservative resistance
Advocacy and strategic planning	Taiwan’s strategies focus on leveraging political support and public awareness	Indonesia emphasizes engaging moderate religious leaders and international support

*Note.* LGBTQ = lesbian, gay, bisexual, transgender, and queer.

## Discussion

The results of this comprehensive analysis of the factors influencing health care staff attitudes toward lesbian, gay, bisexual, transgender, and queer (LGBTQ) individuals in Taiwan and Indonesia emphasize the critical roles of cultural, educational, and legal contexts. The findings underscore the significant impact of demographic factors such as educational level, cultural background, and personal experiences on health care staff attitudes toward LGBTQ individuals. Higher educational attainment among health care professionals was shown to be associated with more positive attitudes, likely due to greater exposure to diverse perspectives and better understanding of LGBTQ issues (Green et al., 2022). Conversely, traditional cultural backgrounds may perpetuate negative biases that impact negatively on the quality of care provided to LGBTQ patients (Mestvirishvili et al., 2017). Moreover, the findings confirm the powerful influence of religious and cultural contexts, particularly in Indonesia, where conservative norms and strong adherence to Islamic teachings contribute to lower acceptance of LGBTQ individuals (Levy & Levy, 2017; Tan, 2024). LGBT identities are widely rejected in Indonesia because conservative Islamic teachings, upheld by major religious organizations such as the Indonesian Ulema Council, frame nonheteronormative orientations as sinful deviations that threaten traditional family structures and social morals (Asbi Malik et al., 2024). In addition, LGBT identities are often viewed as “western imports” that conflict with national and religious values, with religious groups mobilizing political influence to oppose LGBT rights through legislation and public discourse.

In Taiwan, the progressive legal framework supporting LGBTQ rights has fostered a more inclusive health care environment. Also, the legalization of same-sex marriage in 2019 and subsequent policy developments have positively influenced health care practices, encouraging institutions to adopt LGBTQ-inclusive policies and training programs (Drabble et al., 2020; Rhodes et al., 2016). These changes illustrate the potential for legal recognition to drive societal acceptance and improve health outcomes for LGBTQ individuals. In contrast, the restrictive legal environment in Indonesia, characterized by the criminalization and stigmatization of LGBTQ identities, presents significant barriers to inclusivity. Health care professionals in Indonesia often face cultural and religious pressures that hinder their implementation of LGBTQ-friendly practices (Levy & Levy, 2017).

Promoting LGBTQ inclusivity in health care settings, particularly in conservative cultures such as Indonesia, requires a multifaceted approach that both considers local norms and advocates effectively for LGBTQ rights. One strategy shown to be effective is implementing comprehensive training programs for health care providers that focus on LGBTQ health issues and cultural competency. The Harvard Medical School’s Sexual and Gender Minority Health Equity Initiative exemplifies how curricular innovations enhance the understanding and care provided to LGBTQ patients (Keuroghlian et al., 2022). Adapting these programs to align with local cultural sensitivities is crucial and may involve collaborating with local LGBTQ organizations to ensure relevance and respect for cultural contexts (Keuroghlian et al., 2021).

Fostering partnerships between health care providers and LGBTQ communities also represents a crucial strategy for improving health outcomes. Research indicates fostering stigma-free environments in which LGBTQ individuals feel safe and welcomed significantly enhances health outcomes, particularly in areas such as HIV prevention and treatment. Establishing collaborative partnerships that facilitate communication between health care professionals and LGBTQ organizations can help tailor health care delivery to the specific needs of this population. Comprehensive training programs such as those employed in certain advanced medical initiatives exemplify the adoption of culturally competent practices, which are essential to fostering inclusive health care environments (Lin et al., 2017; Shao et al., 2024). In Indonesia, creating safe spaces within health care settings can encourage LGBTQ individuals to seek necessary care. Community outreach programs that educate both health care providers and the public regarding LGBTQ health needs and rights can facilitate this process (Shaikh et al., 2024).

Policy advocacy is also crucial to the effective promotion and adoption of LGBTQ rights within health care systems. Supportive policies such as antidiscrimination laws and inclusive health insurance coverage are associated with better health outcomes for LGBTQ populations (King et al., 2024). In Indonesia, advocating for related policies, while challenging, is essential. Collaborating with international human rights organizations and local advocacy groups can help amplify these efforts and create a more supportive environment for LGBTQ individuals (Tan, 2024).

In summary, promoting LGBTQ inclusivity in health care settings, especially in conservative cultures such as Indonesia, requires a combination of education, community engagement, and policy advocacy. By implementing culturally sensitive training programs, fostering partnerships with LGBTQ communities, and advocating for supportive policies, health care institutions can create a more inclusive environment that ultimately improves health outcomes for LGBTQ individuals. Higher educational attainment and exposure to diversity were found to contribute to positive attitudes (Green et al., 2022), which is consistent with the global literature on LGBTQ inclusivity (Hurd & Li, 2024). The findings of this study underscore the importance of culturally tailored educational programs and collaboration with local LGBTQ communities (Keuroghlian et al., 2021). Policy advocacy remains crucial, especially in conservative environments, to ensure equitable health care access (Eschliman et al., 2023; King et al., 2024).

### Conclusions and Implications for Practice

The results of this study highlight the critical influence of cultural, educational, and legal contexts on health care staff attitudes toward lesbian, gay, bisexual, transgender, and queer (LGBTQ) individuals in Taiwan and Indonesia. The progressive legal context established in Taiwan has fostered inclusivity, while Indonesia continues to face significant barriers due to conservative cultural and religious norms. Strategies to improve inclusivity include implementing culturally sensitive training, fostering community partnerships, and strengthening policy advocacy. These findings inform global approaches to advancing LGBTQ rights in health care. Integrating LGBTQ health topics into medical training can enhance cultural competence and create stigma-free environments, which have been shown to improve health outcomes and advance LGBTQ rights. Future research should explore the long-term impacts of these strategies and consider intersectionality in health care experiences. The findings emphasize the importance of education, community engagement, and policy advocacy in promoting LGBTQ inclusivity. Health care institutions can create more inclusive environments by addressing cultural and legal barriers, ultimately improving health outcomes for sexual and gender minorities globally and providing a roadmap for policy development and advocacy.

### Recommendations and Limitations

The findings of this study support several recommendations to foster more inclusive health care environments for LGBTQ individuals in Taiwan and Indonesia. First, policy enhancement and implementation are crucial. In Taiwan, developing specific policies for LGBTQ inclusivity in health care, including mandatory training programs, is vital to continuing to improve inclusivity in health care settings. In Indonesia, prioritizing comprehensive legal protections supported by international human rights frameworks is necessary. Second, advocacy and public education are essential. Taiwan should continue building on existing support for LGBTQ rights, while Indonesia should engage with moderate religious leaders and civil society to foster acceptance. Continuous research and monitoring of LGBTQ policy implementation will be necessary to identify gaps and assess the effectiveness of interventions.

This study is affected by several limitations. The sample was collected using a convenience sampling strategy, which may limit the generalizability of the findings. Furthermore, the findings are limited by a lack of data on religious affiliation, particularly in light of the known strong influence of religious beliefs on attitudes toward LGBTQ individuals in Indonesia. Future studies should include religious demographics to facilitate a more nuanced understanding of sociocultural influences on health care staff attitudes. In addition, the relatively small and unequal samples recruited in the two countries may affect the robustness of cross-national comparisons. By addressing these limitations, future research may be expected to provide deeper insights and additional culturally grounded strategy suggestions to support LGBTQ inclusivity in health care.
